# The IBER study: a feasibility randomised controlled trial of imagery based emotion regulation for the treatment of anxiety in bipolar disorder

**DOI:** 10.1186/s40345-023-00305-8

**Published:** 2023-07-22

**Authors:** Craig Steel, Kim Wright, Guy M. Goodwin, Judit Simon, Nicola Morant, Rod S. Taylor, Michael Brown, Susie Jennings, Susie A. Hales, Jemma Regan, Michaela Sibsey, Zoe Thomas, Lynette Meredith, Emily A. Holmes

**Affiliations:** 1 Oxford Health NHS Foundation Trust and University of Oxford, Oxford, UK; 2 University of Exeter, Exeter, EX4 4PY UK; 3 University of Oxford, Oxford, UK; 4 Department of Health Economics, Center for Public Health, Medical University of Vienna, Vienna, Austria; 5 Department of Psychiatry, University of Oxford, Oxford, UK; 6 University College London, London, UK; 7 MRC/CSO Social and Public Health Sciences Unit and Robertson Centre for Biostatistics, University of Glasgow, Glasgow, UK; 8 University of Cambridge, Cambridge, UK; 9 University of Reading, Reading, UK; 10 Devon Partnership NHS Trust, Devon, UK; 11 University of Surrey, Surrey, UK; 12 University of East London, London, UK; 13 Department of Psychology, Uppsala University, Uppsala, Sweden

**Keywords:** Bipolar disorder, Anxiety, Emotion regulation, Mental imagery, Psychological intervention, Feasibility

## Abstract

**Background:**

Intrusive mental imagery is associated with anxiety and mood instability within bipolar disorder and therefore represents a novel treatment target. Imagery Based Emotion Regulation (IBER) is a brief structured psychological intervention developed to enable people to use the skills required to regulate the emotional impact of these images.

**Methods:**

Participants aged 18 and over with a diagnosis of bipolar disorder and at least a mild level of anxiety were randomly assigned (1:1) to receive IBER plus treatment as usual (IBER + TAU) or treatment as usual alone (TAU). IBER was delivered in up to 12 sessions overs 16 weeks. Clinical and health economic data were collected at baseline, end of treatment and 16-weeks follow-up. Objectives were to inform the recruitment process, timeline and sample size estimate for a definitive trial and to refine trial procedures. We also explored the impact on participant outcomes of anxiety, depression, mania, and mood stability at 16-weeks and 32-weeks follow-up.

**Results:**

Fifty-seven (28: IBER + TAU, 27: TAU) participants from two sites were randomised, with 50 being recruited within the first 12 months. Forty-seven (82%) participants provided outcome data at 16 and 32-weeks follow-up. Thirty-five participants engaged in daily mood monitoring at the 32-week follow-up stage. Retention in IBER treatment was high with 27 (96%) attending ≥ 7 sessions. No study participants experienced a serious adverse event.

**Discussion:**

The feasibility criteria of recruitment, outcome completion, and intervention retention were broadly achieved, indicating that imagery-focused interventions for bipolar disorder are worthy of further investigation.

## Background

The treatment of bipolar disorder (BD) continues to represent a major challenge (Goodwin et al. 2016). People diagnosed with this disorder suffer from high rates of relapse and suicide (Jamison 2000), whilst development of effective psychological treatments has been limited. Current UK guidelines (NICE 2014) state that the evidence base of psychosocial interventions for BD is mainly of low quality. The range of options, derived from the outcomes of low to moderate quality trials which produced mixed results, includes group interventions, psychoeducation, family-focused therapy, cognitive-behavioural therapy (CBT), interpersonal and social rhythm therapy and integrated cognitive and interpersonal therapy. These treatments mainly target the outcomes of depression and relapse rates.

Anxiety has been neglected as a treatment target within this disorder (Stratford et al. 2015). This is despite evidence that clinical levels of anxiety can persist between acute episodes of mania and depression (Pavlova et al. 2015), and is associated with higher levels of mood fluctuation and a reduced response to mood stabilizing medication (Keller 2006; Otto et al. 2006). New treatments may therefore benefit from targeting specific mechanisms proposed to underlie the inherent mood instability and anxiety within BD (Holmes et al. 2008).

Cognitive-behavioural therapy is based on the premise of working with verbal thoughts expressed in words and forms the basis of one recent approach to working with anxiety in BD (Jones et al. 2018). However, the experience of emotional and intrusive mental imagery as a form of cognition has been associated with a range of mental health problems (Jones et al. 2018), and yet remains a novel treatment target in this group. Although intrusive images are commonly associated with memories, e.g. ‘flashbacks’ within posttraumatic stress disorder, they can also be experienced as ‘flash-forwards’ to emotional events which may happen in the future (Ji et al. 2019; Ivins et al. 2014). People diagnosed with BD are prone to experiencing frequent, intrusive and emotional mental images in this form e.g. an image of attempting suicide (fueling anxiety), or of winning a music prize (fueling elation) (Hales et al. 2011). These images are often reported to be very vivid and have ‘lifelike’ qualities which amplify their emotional impact (Ivins et al. 2014), and therefore represent a target for treatment with the potential to reduce anxiety and improve mood stability (Holmes et al. 2008; Ng et al. 2016; Di Simplicio et al. 2016).

One such recent development in the field is a brief structured psychological intervention which translates experimental work in the area of mental imagery and emotion into a psychological skills training programme to improve the regulation of intrusive and distressing emotional mental images in BD (Holmes et al. 2019). An uncontrolled case series using this approach has produced encouraging results with reduced levels of depression, improved mood stability and a high level of engagement with treatment (Holmes et al. 2016). This study also developed the measurement of mood outcomes by repeatedly capturing mood on a daily basis, over a period of days; thus overcoming typical isolated time point assessments (i.e. on 1 day only), which may not fully capture the inherent mood instability in BD.

The aim of the current study was to assess the feasibility and acceptability of a future definitive trial to evaluate the clinical and cost effectiveness of a brief psychological intervention, here termed Imagery Based Emotion Regulation (IBER), for reducing anxiety within adults with BD. In line with the earlier case series (Holmes et al. 2016), daily mood ratings over 28 days were used to measure mood instability.

As pre-specified in our protocol paper (Steel et al. 2020) the aims were:

1. To inform the recruitment and timeline of a full trial, by establishing the number of participants identified, approached, consented and randomised within a fixed period along with the participant retention rates for follow-up assessment and completion of intervention
2. To inform the sample size estimation of a future trial
3. To refine trial procedures by establishing the acceptability of the trial process to participants including randomisation and participant-perceived relevance and burden of the outcome measure
4. To further assess the acceptability of the treatment and, based on input from trial participants and clinicians, to further refine and develop the treatment manual and the procedures for training, supervising and assessing the competence of trial therapists

## Method

This feasibility study was reported according to the CONSORT 2010 guidelines for randomised pilot and feasibility trials (Butcher et al. 2022). The full trial protocol detailing study design and methods has been published (Steel et al. 2020) and is summarised below.

### Trial design

A feasibility study with a two-arm randomised parallel controlled trial conducted in two UK centres: Berkshire Healthcare NHS Foundation Trust (BHFT) and Oxford Health NHS Foundation Trust (OHFT) (combined as one site) and Devon Partnership NHS Trust (DPT). The study was approved by the NHS Research Ethics Committee (reference 18/SC/0164). The study aimed to recruit 60 participants randomly allocated 1:1 to an intervention plus treatment as usual (IBER + TAU group) or TAU alone (TAU group).

### Participants

Referrals were accepted from in-patient services, primary and secondary care and self-referral. Referrals were sought from people aged 18 or above who presented with symptoms consistent with a DSM-V diagnosis of bipolar disorder (I, II or otherwise specified) assessed using the Structured Clinical Interview for DSM-5 (SCID) (First et al. 2015; American Psychiatric Association 2013). Potential participants were required to have a sufficient understanding of English in order to be able to engage in the study, and to exhibit at least a mild level of anxiety by scoring 5 or above on the GAD-7 (Spitzer et al. 2006). Exclusion criteria were (i) a current episode of mania or depression (ii) unable to provide informed consent (iii) acute suicide risk (iv) DSM-5 diagnosis of substance use or alcohol use disorder, moderate or severe, assessed using the SCID (v) a change in medication within 3-months prior to randomisation or (vi) currently engaged in a psychological intervention.

### Randomisation and blinding

Randomisation was stratified by trial site and minimised on medication status (in receipt of prescribed mood stabilisers vs. not) and anxiety severity [GAD-7 > 14 (severe anxiety) vs GAD-7 ≤ 14]. Web-based randomisation was conducted independently, by the Thames Valley Clinical Trials Unit (TVCTU), using randomised permuted blocks.

Group allocation was transparent to the participant, trial manager and trial therapists whilst the researchers responsible for collecting assessment data remained blind to group allocation. The trial adhered to procedures designed to maintain separation between research staff who obtained measures and clinical staff who delivered the intervention. This included the use of separate offices, separate booking systems when seeing participants and separate agendas within team meetings. As all follow-up assessments were done online or via post, blind-breaks did not occur during assessments. Where an allocation was revealed to an assessor at any point during the study, masking was maintained through a new assessor being the point of contact thereafter.

### Interventions

Imagery Based Emotion Regulation (IBER) is a structured individual psychological intervention consisting of up to 12 sessions to be delivered within 16 weeks. The intervention targets maladaptive mental imagery. An in-depth assessment phase leads to the identification of a target image or images co-identified and formulated by the client and therapist as impacting on anxiety and mood instability. In the active treatment phase visual imagery techniques are applied to the formulated target. The final skills consolidation phase aims to embed strategies in a memorable format for clients to access easily in future. IBER was informed by, though not the same as, the manual we have developed on the basis of our previous work (Holmes et al. 2019, 2016; Hales et al. 2018). Further details of the three stages are given below:

*Assessment:* This stage occurs over 4 sessions and includes assessment of current positive coping strategies, ability to recognise prodromes of mood episodes and, where necessary, the development of a crisis management plan. This is followed by the identification of current emotional mental images impacting on anxiety and mood instability, and the creation of an individualised formulation which includes imagery-related beliefs and responses.

*Treatment*: Four theoretically informed mental imagery-based interventions have been developed as detailed below. The individual formulation created in the assessment phase maps out images to target in the intervention, and the individual treatment approach follows from this.

Typical images worked on in therapy included intrusive images related to the client’s bipolar disorder, for example, images of being very depressed. Often these were associated with a sense of fear and hopelessness and the meaning “I will end up feeling like that again and won’t be able to cope”. Clients also frequently worked on modifying anxious images about the future, including distorted images of themselves and others in social situations. These images had underlying meanings such as: “I am not like other people”, “I am not accepted”.

Imagery-based intervention techniques are used in isolation or in combination.

(i) Imagery Rescripting (IR) involves assisting people to transform maladaptive or distressing imagery into more functional, benign imagery, thereby updating its underlying meaning. Although adapted from the approach with the same name used for treating traumatic memories (Arntz 2012), here IR was not limited to working with memories but also to modify simulated images of the future. IR is typically adopted when the participant is mainly troubled by one or two repetitive images.
(ii) Metacognitive Techniques aim to reduce the “power” of an image by changing how a client relates to the image. The strategies reinforce an image is “just an image” and not real. Thus, the client does not need to pay attention to the image. Instead, they should direct their attention outside of the image. Such strategies are used with the majority of participants in combination with other techniques.
(iii) Positive Imagery Techniques help participants to generate mood-enhancing or soothing imagery which holds a helpful and adaptive meaning for the client. Imagery of this type is frequently lacking in clients with BD. Positive imagery may be used to induce a sense of well-being, act to bolster self-esteem, or encourage the client to move in the direction of desired goals.
(iv) Imagery-competing Tasks implement concurrent visuospatial activities (such as the computer game Tetris) to reduce the intensity and/or recurrence of problematic imagery. This approach has been used in studies aimed at reducing the frequency of traumatic intrusions (Iyadurai et al. 2020), however in IBER these techniques were mainly used to reduce the impact of images (for example at night when imagery was disrupting sleep) and were always used in combination with one or more of the other imagery techniques detailed.

*Skills consolidation*: skills that have been learnt during treatment are consolidated into an action-plan that the participant can implement This is documented as a personal video designed by the client which captures the action-plan in video film images in addition to words.

The intervention was delivered by four clinicians; all clinical psychologists experienced in using CBT. Training consisted of a two-day programme, and supervision was provided by team members (SH, KY) with relevant expertise both in the intervention and the patient group. Sessions were recorded where the patient gave consent. Adherence to treatment protocol was monitored through the use of a bespoke measure developed by the trial team. The measure consisted of a checklist tailored for each phase of the IBER treatment. Ratings were made for both specific items necessary for the particular phase of treatment (e.g. “helps the client elucidate imagery or other co-morbidities impacting on anxiety”) and general competencies (e.g. “therapist displays a curious stance”). Ratings were made on a 4-point Likert scale from 0 (not adherent) to 3 (good quality and adherent). If an item was rated 2 or above this indicated that the work was of good enough quality to be adherent. Random sessions recorded from 20% of treatment cases were assessed for adherence by an external rater who was an expert in the intervention.

Both groups received TAU which was delivered by mental health professionals from within the NHS Trusts and was based on local protocols. All treatment was recorded as part of the amended Client Service Receipt Inventory (Simon and Mayer 2016) used for the collection of health and social care data and typically included medication and contact with psychiatrists and community psychiatric nurses, while information on the IBER intervention was recorded as part of the trial therapist diaries.

### Outcome assessment

Assessments were conducted by graduate psychologists at baseline (prior to randomisation), 16-weeks (end of treatment) and 32-weeks follow up post-randomisation through self-report questionnaires, completed predominantly via a secure online questionnaire system (ePRO^®^, P1vital Products Ltd.). A small number (n = 3) of participants completed paper questionnaires which were returned by post. Potential participants must have completed all baseline assessments, and at least 23 out of the 28 daily mood monitoring measurements conducted prior to baseline (see below) in order to meet inclusion eligibility.

The primary outcome was anxiety as measured by the GAD-7 (Spitzer et al. 2006) at end of treatment. Secondary outcomes were depression, as measured by Quick Inventory of Depressive Symptomatology–Self Report (QIDS-SR) (Rush et al. 2003), and mania as measured by the Altman Self-Rating Scale for Mania (ASRM) (Altman et al. 1997). Each of these three outcomes (anxiety, depression and mania) were measured by administering self-report questionnaires on four separate occasions—one week apart—covering a 28-day period, with the mean value captured as the reference point. Baseline data covered the 28-days prior to randomisation, and follow-up data covered the 28-days after each follow-up assessment due date (i.e. starting at 16-weeks and 32-weeks post randomisation).

Mood stability was measured by participants rating (0–6) how anxious, elated, sad and angry they felt on a daily basis over the same 28-day period at baseline, end of treatment and follow-up (Tsanas et al. 2016).

Patients’ health-related quality of life was measured by the EuroQol EQ-5D-5L (The EuroQol Group 1990), whilst general wellbeing was measured by the ICECAP-A (Al-Janabi et al. 2012) and OxCAP-MH (Simon et al. 2013) instruments. Health care resource and costs were measured using the Health Economics Questionnaire (HEQ) (Simon and Mayer 2016). All health-related measures were collected every 28-days from the start of the trial, until the follow-up assessment.

All serious adverse events were documented throughout the trial and reported to the Data Monitoring and Ethics Committee, where the independent chair determined whether the event was attributed to the delivery of the intervention. Non-serious adverse events were also recorded.

After trial completion all participants were posted a questionnaire to assess their experiences of both the trial procedures and intervention. A sub-sample of those allocated to the intervention were invited to take part in an in-depth interview to discuss their experiences of IBER treatment.

### Data analysis

The sample size of 60 participants was sufficient to achieve the feasibility objectives. The data analysis was presented on a descriptive level. The study reports recruitment, study attrition, and intervention (IBER + TAU) completion (≥ 50% of sessions attended) and completion of outcome, where appropriate with 95% confidence intervals. Mean and standard deviations for all outcomes are reported for both study arms at baseline, 16 and 32 weeks, between group differences and 95% CIs reported.

Mood variability is quantified using the standard deviation Root Mean Squared Successive Differences (Altman et al. 1997) for each of the four daily mood measures items.

Feasibility criteria for a full trial assessed during this study [as published in the protocol paper (Steel et al. 2020)] were (i) overall recruitment at ≥ 80% or above within the 12-month recruitment period i.e. ≥ 48 participants recruited (ii) 32-week follow-up data from ≥ 80% of participants on all outcomes (iii) ≥ 80% of participants attend at least 50% of the possible sessions) (iv) no serious negative consequences (serious adverse events) associated with trial or intervention participation.

## Results

### Sample characteristics

**Fig. 1:**
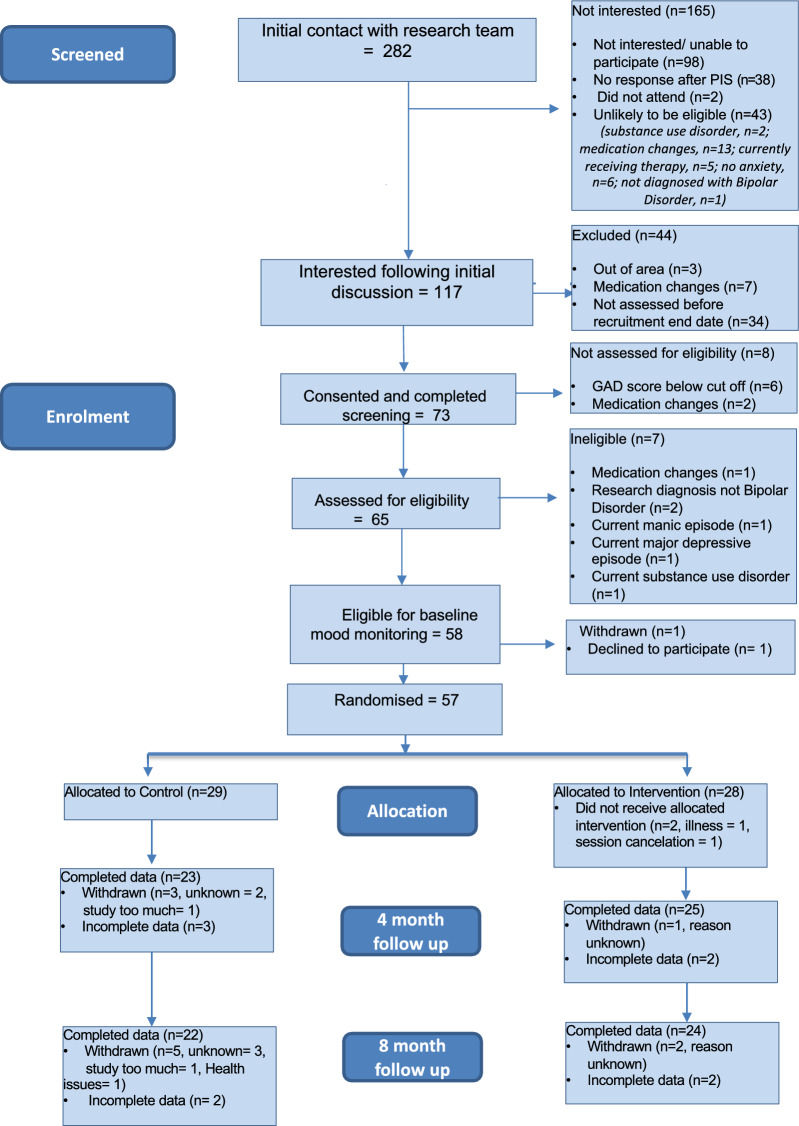
Consort Diagram

In total 282 referrals were received for the present study of whom 73 gave their written and informed consent and were assessed for eligibility using the SCID (First et al. 2015) and GAD-7 (Spitzer et al. 2006) (see Fig. 1). Of these referrals, 57 were eligible and randomly assigned to either the IBER group (n = 28) or treatment as usual (n = 29) (see Fig. 1). Forty-six participants presented with at least one anxiety disorder (Social Anxiety Disorder = 27; Generalised Anxiety Disorder = 26; Agoraphobia = 19; Posttraumatic Stress Disorder = 19; Panic disorder = 18; Specific phobia = 13 and Obsessive Compulsive Disorder = 9). Thirty of the 57 participants were recruited from DPT whilst 27 were recruited from BHFT and OHFT.

The group predominantly identified as White British and female and were prescribed mood stabilisers. Approximately half were in employment. There were no significant group differences within the demographic variables reported (see Table 1).

**Table 1:** Baseline characteristics of the sample

	IBER (n = 28)	TAU (n = 29)	Total (n = 57)
Demographics			
Mean age in years (SD)	46.0 (12,2)	45.5 (12.8)	45.7 (12.4)
Female (%)	64.3	72.4	68.4
White british (%)	82.1	93.1	87.7
Age left formal education	18.1 (3.7)	17.0 (1.9)	17.6 (3.0)
Currently employed (%)	50.0	55.1	52.6
Primary diagnosis			
Bipolar I disorder	53.6	44.8	49.1
Bipolar II disorder	46.2	55.2	50.9
Psychiatric history			
Prior psychiatric hospitalization (%)	64.0	65.4	64.7
Mean number of prior admissions	2.1 (1.6)	4.2 (5.4)	3.2 (4.1)
Mean age at first contact with mental health services	25.6 (11.3)	25.3 (11.5)	25.5 (11.3)
Prescribed mood stabilisers (%)	88.0	85.2	86.5

### Recruitment and retention

Overall recruitment reached 57 participants (95% of target), with 50 participants (83% of target) being recruited during the first 12 months, i.e. just over two participants per month per site during this initial period. Comprehensive 32-week follow-up data from the main outcomes (including the HEQ resource use measurement questionnaire) was obtained from 46 (81%) of the randomised participants, and from 35 (61%) participants for the daily mood monitoring measures. Of the 28 participants who were allocated to IBER, 27 (96%) received a full dose (7 sessions or more; mean number of sessions = 10.5 (*SD* = 1.99) of the treatment.

### Full trial sample size

The estimated sample size required for a full RCT, is 39 participants per group, based on the GAD-7 (Spitzer et al. 2006) standard deviation from the current study, obtaining a mean between group difference of the minimum clinically important difference of ≥ 4 points and assuming 20% attrition (power = 0.9, alpha = 0.05).

### Trial acceptability and process refinement

Three serious adverse events were recorded during the trial. None of which were deemed as associated with the intervention or trial procedures. The randomly selected treatment cases were all rated as adherent to the treatment protocol. Thirty-three (58%) participants returned a post-trial exit survey at the end of follow-up data collection. Of these, over 90% endorsed being at least ‘moderately satisfied’ with the clarity of information provided, assessment procedures, assessment burden and overall trial experience. Participants’ experiences of the IBER intervention obtained through qualitative interview will be reported in a separate publication and discussed in relation to future iterations of the intervention.

### Outcome measures

Primary and secondary outcome data at each assessment point across both conditions are shown in Table 2. As the study was not powered for significance testing, the outcomes at end of treatment and follow-up are presented as between group difference and Cohen’s effect sizes (Table 3).

**Table 2:** Patient-related outcomes at baseline and follow-up: descriptive summary

	Baseline mean (SD) N		16-week follow up mean (SD) N		32-week follow up mean (SD) N	
	IBER	TAU	IBER	TAU	IBER	TAU
Generalised anxiety disorder assessment (GAD7) *Range 0 to 21 (minimal anxiety to severe anxiety)*	8.2 (4.8) 28	8.8 (4.2) 29	5.7 (4.8) 24	8.4 (5.9) 22	6.8 (5.7) 23	7.8 (5.3) 22
Quick inventory of depressive symptomatology–self report (QIDS-SR) *Range 0 to 37 (higher score indicates higher depressive symptoms)*	9.6 (5.2) 28	9.6 (4.7) 29	6.8 (4.0) 25	10.0 (5.6) 22	7.7 (5.0) 23	9.6 (5.6) 22
Altman Self-Rating Scale for Mania (ASRM) *Range 6 to 20 (higher score indicates increased manic symptoms)*	3.5 (3.6) 28	2.1 (2.1) 29	2.4 (2.3) 25	1.5 (2.0) 23	2.9 (4.2) 23	1.5 (2.1) 22
EuroQol EQ-5D-5L—index *Range -0.594 to 1.0 (0 indicates death and 1 indicates perfect health)*	0.66 (0.25) 28	0.62 (0.31) 29	0.73 (0.25) 25	0.61 (0.29) 23	0.71 (0.62) 25	0.67 (0.33) 23
EuroQol EQ-5D-5L—VAS *Range 0 to 100 (0 indicates death and 100 indicates perfect health)*	59.5 (21.2) 28	55.6 (21.1) 29	64.7 (24.6) 25	61.9 (22.4) 23	62.6 (22.0) 25	61.0 (19.5) 23
ICEpop CAPability measure for Adults (ICECAP-A) *Range 0 to 1.0 (0 indicates no capacity and 1 indicates full capacity)*	0.65 (0.21) 28	0.70 (0.23) 29	0.77 (0.69) 25	0.67 (0.26) 23	0.75 (0.22) 25	0.74 (0.19) 23
Oxford CAPabilities questionnaire-Mental health (OxCAP-MH) *range 16 to 80 standardised to 0 to 100 (0 indicates no capability and 100 maximum capability)*	56.0 (10.3) 27	59.0 (8.4) 29	61.7 (8.9) 25	58.7 (8.2) 23	57.7 (9.4) 25	57.2 (8.2) 23

**Table 3:** Patient-related outcomes at follow-up

	Between group difference^a^ (TAU-IBER) 16-week follow up		Between group difference^a^ (TAU-IBER) 32-week follow up	
	Mean (95% CI) N	Effect size^b^	Mean (95% CI)	Effect size^b^
Generalised Anxiety Disorder Assessment (GAD7) *Minimal improvement: 4 points *reduction (Loussaint et al. 2020)	1.4 (− 1.3 to 4.0) N = 44	0.16	0.2 (− 2.4 to 2.8) N = 44	0.02
Quick Inventory of Depressive Symptomatology–Self Report (QIDS-SR) *Minimal improvement: 28.5% *reduction (Masson and Tejani 2013)	2.8 (0.5 to 5.1) N = 47	0.29	1.7 (− 1.0 to 4.4) N = 45	0.18
Altman Self-Rating Scale for Mania (ASRM) *Minimal improvement: 5.4 *reduction (Altman et al. 2001)	− 0.5 (− 1.6 to 0.6) N = 48	− 0.18	− 0.5 (− 2.3 to 1.3) N = 45	− 0.18
EuroQol EQ-5D-5L, index *Minimal improvement: 0.05 *increase (Payakachat et al. 2015)	− 0.07 (− 0.18 to 0.04) N = 48	− 0.11	− 0.002 (− 0.14 to 0.14) N = 48	− 0.003
EuroQol EQ-5D-5L, VAS *Minimal improvement: 5 *increase (Goranitis et al. 2016)	− 3.5 (− 15.5 to 8.3) N = 48	− 0.06	− 2.0 (− 13.3 to 9.3) N = 48	− 0.03
ICEpop CAPability measure for Adults (ICECAP-A) *Minimal improvement: 0.3 *increase (Goranitis et al. 2016)	−0.12 (− 0.21 to -0.03) N = 48	− 0.18	− 0.03 (− 0.13 to 0.06) N = 48	− 0.04
Oxford CAPabilities questionnaire-Mental Health (OxCAP-MH) – 117 *Minimal improvement: 6.47 *decease (Vergunst et al. 2017)	− 4.8 (− 8.5 to -1.1) N = 47	− 0.08	− 3.5 (− 7.4 to 0.4) N = 47	− 0.07

^a^Adjusted for baseline value and stratification variable of trial site (Devon or Berkshire) and minimisations variables of medication status (i.e. prescribed mood stabilisers) and anxiety severity (severe anxiety being a score above 14 on the GAD7)

^b^mean between group difference/pooled baseline SD

The only clinical outcome to reach the threshold of a minimal clinically important difference was the depression score at end of-treatment. Effect size outcomes for all measures were either small or negative. The mood stability outcomes of the twenty-eight days of daily mood monitoring over the three assessments are presented in Table 4.

**Table 4:** Mood Stability as measured by Root Mean Squared Successive Difference at baseline and follow-up: descriptive summary

	Baseline mean (SD) N		16-week follow up mean (SD) N		32-week follow up mean (SD) N	
	IBER	TAU	IBER	TAU	IBER	TAU
Anxious	1.34 (0.53) 24	1.41 (0.53) 24	0.92 (0.52) 25	1.27 (0.53) 20	1.03 (0.45) 20	1.21 (0.50) 20
Elated	1.12 (0.75) 24	1.24 (0.64) 24	0.85 (0.53) 25	0.95 (0.55) 20	0.90 (0.65) 20	1.04 (0.56) 20
Sad	1.25 (0.60) 24	1.46 90.51) 24	0.95 (0.59) 25	1.23 (0.51) 20	0.93 (0.46) 20	1.19 (0.38) 20
Angry	1.10 (0.61) 24	1.43 (0.47) 24	0.82 (0.66) 25	1.04 (0.57) 20	0.60 (0.51) 20	1.16 (0.69) 20

## Discussion

The current study aimed to assess the feasibility of a full trial to evaluate the effectiveness of IBER as a treatment for anxiety in people diagnosed with bipolar disorder. Feasibility criteria were broadly achieved, including recruitment (> 80% at 12-months), outcome completion (> 80% at 32 weeks follow up), and intervention participation (> 80% attended > 5 sessions). The majority of participants were at least moderately satisfied with the experience of being a trial participant, and there was an absence of trial and intervention related serious adverse events. Recruitment was established within three UK NHS Trusts at a recruitment rate of just over 4 participants per month in the first 12-months. Our experience in conducting this study informs us that recruitment could be enhanced through establishing links with GP practices at the early stage of a trial.

Overall retention in the trial, both for treatment and assessments, and reached the feasibility thresholds set at the start of the study. Treatment retention was particularly high, with over 96% allocated to receive the IBER intervention seven sessions or more. This level of engagement compares favourably to recent comparable trials, e.g. 50% attending at least 50% of mandatory sessions [ThrIVe-B programme (Wright et al. 2021)] and 59% attending 9–10 sessions within 16 weeks [CBT for anxiety in bipolar disorder (Jones et al. 2018)].

All of the clinical and health outcome measures collected at the end of treatment and at the 32-week follow-up reached the retention threshold set for feasibility. Delivering these assessments online facilitated engagement for most participants. However, this must be complemented with close monitoring of those participants who did not engage with the online process, and direct contact to facilitate the process. One area which requires attention is the daily mood measures, which fell below the threshold level. This trial, and current trends in the field, are motivated by the significant limitations of using fixed time point assessments for a group of people who inherently experience frequent mood fluctuation. With mood instability being an important treatment target, it is important to allocate increased resources to ensuring these data are collected in a convenient and acceptable way and with the appropriate prompting and support. Compliance to daily mood monitoring may be increased by promoting the benefits of taking autonomy over self-assessment, as demonstrated in existing literature in the field.

The safety of the intervention is demonstrated via the lack of any associated serious adverse events. The high level of engagement with clinical sessions is a good indicator of treatment acceptability. Further details on participants’ views and experience of IBER based on qualitative interviews will be reported elsewhere.

As with all feasibility studies, the current trial was not powered nor designed to test clinical effectiveness. Baseline anxiety levels in the current study were comparable to those within the previously conducted case series (Holmes et al. 2016). As would be expected, observed effects were lower due to adopting a more robust design, most notably the use of a blind assessed control group. However, most outcomes are in favour of the intervention arm of the trial. The decrease in effect size between end of treatment and follow-up assessments indicates that booster sessions maybe useful. This was corroborated by participants during the qualitative interviews.

Whilst our sample were not recruited during a major mood shift, they were experiencing lower-level mood instability which is both distressing and a valid treatment target (Stratford et al. 2015; Pavlova et al. 2015; Keller 2006; Otto et al. 2006; Holmes et al. 2008). The basis of our study is that IBER targets anxiety and mood instability in order to reduce distress and improve quality of life. There is also a rationale that improving skills in regulating daily emotions may enable greater control over potential larger emotional change. However, it was not the aim of our feasibility to test this relationship. A future full trial would benefit from a longer follow-up period to assess the impact of IBER on relapse rates. Any future full RCT will also need to account for the potential for non-specific therapy factors to contribute to any observed difference in group outcomes. This will include the close monitoring of all contact time via any health intervention.

This paper demonstrates a robustly conducted study which provides a strong basis for further research utilising a full trial design. Given the current lack of evidence-based psychological interventions for people diagnosed with bipolar disorder, the lack of treatment for bipolar anxiety and the favourable engagement with the imagery-focussed intervention by participants, the current intervention appears worthy of further investigation.

## Data Availability

Not currently applicable. The datasets generated and/or analysed during the current study will be available from the corresponding author on reasonable request following the publication of results. Details of the therapy can be found in Holmes, E.A., Hales, S.A., Young, K. & Di Simplicio, M. (2019). *Imagery-Based Cognitive Therapy for Bipolar Disorder and Mood Instability.* New York: Guilford Press. ISBN 9781462539055.
